# Life in vaccine science – a conversation with Stanley Plotkin at the 4th Conference on Vaccines in Dubrovnik, Croatia, September 2017

**DOI:** 10.2807/1560-7917.ES.2018.23.17.18-00147

**Published:** 2018-04-26

**Authors:** Sanjin Musa

**Affiliations:** 1 Public Health Institute of the Federation of Bosnia and Herzegovina, Sarajevo, Bosnia and Herzegovina

To address a concern that a lack of evidence-based information about vaccines among healthcare providers and the public could undermine the future of the immunization programs, the European Society of Clinical Microbiology and Infectious Diseases (ESCMID) organised the 4th Conference on Vaccines in Dubrovnik, Croatia, from 8–10 September 2017. The conference was titled ‘New and old diseases in children and adults – unmet needs’. Leading experts were brought together to present and debate the most recent data about vaccines for the purpose of developing strategies to ensure sustained confidence in immunisation programmes and the protection of future generations from vaccine-preventable diseases.

One of the presenters was Stanley Plotkin, a prominent figure in the history of vaccinology, whose work on vaccine development has led to a significant reduction in morbidity and mortality from infectious diseases in the second part of 20th century. Plotkin, 85 years of age at the time of the interview, started his career during ‘the golden age of vaccinology’. This interview with him was conducted at a time when ongoing measles outbreaks in the WHO European Region had caused 35 deaths in the past 12 months [1]. The following is an edited transcript of the interview. Editorial insertions are within square brackets.

**Sanjin Musa (SM):** You graduated from the State University of New York Medical School in 1956. What led you to decide to focus on research and discovery?

**Stanley Plotkin (SP):** So, I have already published one article answering that question in *The Pediatric Infectious Disease Journal*, years ago. It was titled, if I recall correctly, ‘The late sequelae of Arrowsmith’. The reason for the title was that when I was 15, I read two books. One was titled *Microbe Hunters* by Paul de Kruif, and the other was a novel called *Arrowsmith* by Sinclair Lewis. Arrowsmith was the name of the character in the novel who was a physician that had been through a number of things in the novel, but eventually ended up as a vaccine developer. It was reading those two books that made me decide to follow a career in vaccines.

**SM:** You have lived during a time when children commonly experienced childhood diseases like polio, diphteria and rubella. In the middle of the 20th century, poliomyelitis was an epidemic disease. The first successful attempt of vaccine development by attenuation of polio virus was made by Hilary Koprowski during his work at the Lederle Laboratories. At the time you started to work in the Wistar Institute, it was under the leadership of Hilary Koprowski and the Wistar Institute became a leader in vaccine research. Can you tell me about that time?

**SP:** First of all, I went into the US [United States] public health service as a member of the epidemic intelligence service. At the end of the training, they offer you a job somewhere, either within a state health department or somewhere else. One of the positions that was open, when it was my turn, was a position doing research on anthrax. This was located at the Wistar Institute and because I was reading papers, I read papers by Hillary Koprowski. They were very interesting and included, of course, papers on polio. I had also read that he was coming to Wistar to be director, so I asked for the position to work on anthrax thinking that at Wistar, I might be working with Koprowski. Indeed that happened. When I arrived, I went to see him, and he had just arrived. I asked him if I could work in his virus lab and he agreed. So I did anthrax, but I also did polio.

Hillary was an extremely interesting person. He was born in Poland. When the Nazis came, he escaped Poland and went first to Italy. He was a brilliant pianist and had studied at the Academy Santa Cecilia in Rome. Of course, he couldn't stay in Italy, so then he emigrated to Brazil, where he took a position in the Pan American Health Organization. From Brazil, he took a job at Lederle and eventually became director at Wistar.

So Hillary was a very learned person. He knew a lot about art, music, history. It was fascinating to work with him. If you look at some of his papers, he has quotations from ancient literature. He was someone whom I admired. He was very open to letting me work in his lab. That is how I started to work on polio, but I also learned a lot about virology in general. One other thing that I would say about Hillary was that as a director of the institute, he was a genius in choosing people and letting them to do their things, but at the same time, giving them suggestions. So it was a wonderful place to work for a young person.

**SM:** Finally, in the late 1950s, the decision was made that the Sabin strains would be the best option to control poliomyelitis...

**SP:** There were three laboratories working on the development of a polio vaccine, Sabin was one [Albert Sabin worked at Cincinnati Children's Research Foundation, the University of Cincinnati], Wistar was a second and Lederle continues to work on attenuation of polio virus. Two things made a difference. One was that Hillary never obtained a really good type 2 strain. He obtained one from a researcher named Cox [Herald Cox] who had essentially cultivated a type 2 strain from natural polio. A second problem was that, I gave Albert Sabin a great deal of credit for his laborious, very thorough work on attenuation. The result was that, when three sets of strains were tested by the Division of Biological Standards in the US, the best neurovirulence data were from the Sabin strain. As you know, there are three types of polio. Type 1 strain, which I worked on also, by Hillary was just as good as Sabin type 1 strain, if not even better. But the Type 2 strain was not as good. The Type 3 strain was about the same, but maybe Sabin’s was slightly better. The result was that the Sabin strains were licensed in the US. But there was another issue that was very important. That was that Sabin was born in Russia and he spoke Russian. Actually, Hillary also spoke Russian, but Sabin became friendly with a Russian virologist. He was able to get their agreement to test Sabin strains in Russia, and they did. They reported that they vaccinated 10 million people and nobody had turned a hair. In retrospect, I think that was probably not true because today we know that there is reversion of attenuation and so forth, but the results were the first large-scale results in the world. Meanwhile, Hillary had trouble finding a place to test his vaccines. He tested them somewhat in [what was at the time] the Belgian Congo [designation from 1908–1960, today Democratic Republic of the Congo], where I went to help, and also in Poland. That was not as impressive as the results with Sabin’s strains. Sabin’s strains were adopted by the US, and then other countries adopted them. Hillary’s strains were used in Poland for some period, but eventually replaced by Sabin strains because vaccine manufacturers adopted Sabin strains. This is essentially what happened.

**SM:** Isolation of the rubella virus and early vaccine development occurred at the start of an epidemic of rubella in Europe and the US in 1963 and 1964. The vaccine strain RA 27/3 that you developed became the accepted rubella strain used throughout the world, mostly in the combined measles, mumps, rubella (MMR) vaccine. Can you tell us something about the race for a vaccine?

**SP:** This was also an interesting story. First of all, as you said, the epidemic started in Europe. It so happened that I spent a year in London at Great Ormond Street Children’s Hospital, and there was a virus laboratory where I worked with an Englishman named Alistair Dudgeon who was also interested in rubella.

We began to see, in the spring of 1963, patients with rubella, and that was impressive. Then the epidemic spread to the US. Meanwhile, I’d gone back to the US and at just about that time, two laboratories developed methods to isolate the rubella virus. Indeed, I started to work on the rubella virus in London at Great Ormond Street Children’s Hospital using the isolation technique in African green monkey kidney cells, but then I went back to Wistar. It seemed to me that what I learned about attenuating polio viruses could be applied to rubella; in particular, adaptation at low temperatures. But then, a second important factor was that at Wistar at about that time, two people, Leonard Hayflick and Paul Moorhead, had developed ways of cultivating human fetal diploid cells. That was very important, because these were normal cells that could not go on forever, unlike continuous cell line. The issue of cancer, et cetera, was obviated by using the fetal cells, so I decided to adapt the rubella virus to the fetal cells, and to attenuate them by classical plaquing and low temperature growth. Eventually, testing in humans showed that it was attenuated. But I was not the only one working on rubella and what happened was that workers at the Division of Biological Standards developed their own vaccine in the African green monkey kidney. They obviously had an advantage since they came from the licensing organisation, and Merck adopted their strain.

**SM:** It was the credit of Maurice Hilleman to replace the HPV77-DE5 strain with RA 27/3 strain in MMR vaccine.

**SP:** That’s right. Merck used HPV77, but studies showed that first of all, RA 27/3 was less reactogenic and secondly, that it produced better immune responses. At a certain point, Hilleman decided that he would replace HPV77 with RA 27/3. In addition, the Welcome Laboratory in England was still producing vaccines, and they adopted RA 27/3 from the beginning. So actually, RA 27/3 was used in Europe before the US. But once Merck switched, essentially there was no other manufacturer. GSK [GlaxoSmithKline] also adopted RA 27/3. Eventually, all manufacturers were using RA 27/3.

**SM:** The use of human diploid cells as a cell population of choice, and development of WI-38 and MRC-5 was controversial?

**SP:** It was controversial for two reasons. One was that there was this fear that in human cells, there might be some hidden virus that would cause problems. You have to remember that at about the same time, SV40 was discovered in monkey cells. Since that was surprise, people were afraid that the same thing might be true for human cells. A second issue was a religious issue. Fetal cells had been isolated from an aborted fetus in Sweden, and since it was an aborted fetus, there was, let’s say, some issues with the Catholic Church.

Going back to the first issue, a lot of work was done to show that the cells do not have any contaminating viruses, and indeed were, for that reason, safer than many other cells being used to make vaccines. The second issue, as far as the religious issue was concerned, the church decided that the only one who had sinned was me; that people who used the vaccine were free of sin because they were just using something that somebody else had developed. So, those were the controversies.

**SM:** You worked on the development of several other vaccines...

**SP:** ...rotavirus, varicella, rabies, CMV [cytomegalovirus].

**SM:** Looking back, what are your thoughts on the phenomenon of vaccine hesitancy or the broader postmodern medical paradigm which questions the legitimacy of science?

**SP:** As far as vaccine hesitancy is concerned, it is a complex issue because people react to fears. Fear is a very great motivator. So if you live in India, you are threatened constantly by infectious diseases and so you are afraid of diseases, and if somebody comes along with a vaccine, your reaction is to say, ‘oh great, I can protect myself against this disease.’ Conversely, if you live in countries in Europe or if you live in the US and those diseases have largely disappeared because of vaccination, you say to yourself, ‘somebody told me that vaccine can cause x, y or z, and therefore, why I should I take that risk?’ Not only that, but other people are being vaccinated so let them take the risk. Now, of course that is not an ethical point of view, but nevertheless, people can adopt it. It is essentially fear that drives that vaccine hesitancy. It is also misinformation. Unfortunately, we live in an age where lies can travel as fast as truth, and they do. You go on the web…in the US now, we are talking about fake news. People just invent things. I really feel that, what is his name...the guy who published on autism and measles? Wakefield. [..] His activities have influenced people to risk disease and death.

**SM:** What was your reaction to the Andrew Wakefield study and its subsequent reception around the world?

**SP:** It so happened, when Wakefield came out with his paper, that I was working for Sanofi Pasteur and they asked me to read the legal briefs because there were legal cases coming up in the UK [United Kingdom], and I did. I am convinced from what I read, from the testimony of people in his laboratory, for example, that those results were all false. They were just made up essentially. So, I believe that what he has published is untrue and of course, the paper was retracted and he is still spreading false information. But the US is a free country. He is now in the US, in Texas and other places. There was an outbreak of measles in Somali immigrants in Minnesota and he had gone to Minnesota to talk to Somali immigrants to convince them not to be vaccinated…and now we have a very significant outbreak of severe measles in Somali immigrants, and couple of deaths. [..]

**SM:** What we are seeing today is that certain advocacy groups and public individuals, would like to erode the public’s trust in the authority of science.

**SP:** Here, I have to get philosophical. The scientific approach to data is different from what may be called the common approach. Scientists know that often there are data that say one thing and data that say another thing because when you collect data, you are collecting points, numbers, and just by chance, numbers may say one thing and numbers may say another thing…and you look at the overall result and you say the overall result shows 95% certainty that x equals y, whatever. That is not a common sense approach. A common sense approach is if something bad happens after something else there must be relationship, and unfortunately, most people do not understand chance and that is the problem. So, if something bad happens to an individual who received a vaccine containing thiomersal, common sense tendency is to say the cause was thiomersal. [..] Now there was also, because I lived through this, there was also something that happened which I think was, in a way, unfortunate. That is, when the controversy arose, that was back in the 1990s, manufacturers decided to remove thiomersal from most of the vaccines, and I think, in a way, that was a mistake. I can understand why they did it, but on the other hand, it implied that the critics were right and that thiomersal is not safe. In other words, the vaccine community reacted by trying to remove any worry. Maybe that was right thing to do, but on the other hand, it gave the impression that there was a problem.

**SM:** In current mainstream media, stories on so-called ‘vaccine wars’ are frequent.

**SP:** What the press does, and I can understand this, they always feel that they have to present both sides of the story and give them equal value. It means, no matter how crazy you are, you can get a hearing.

**SM:** In 2017, the 7th edition of *Plotkin’s Vaccines*, a reference book in the field of vaccinology, was published. You are the editor, together with Walter Orenstein, Paul Offit and Kathryn Edwards. When and why did you have the idea to compile this book?

**SP**: The first edition was in the 1980s and my motive was very simple. One, I felt that there was now a field of study that was not infectious diseases. It derived from infectious diseases, but it was no longer classical infectious diseases. It was not really immunology, although immunology is of course the basis of vaccines. Vaccinology was now a field itself, and did not have a textbook. I felt that it was time to show that, indeed, it is a separate field and that it needed a source of information where you could find answers to questions about vaccines.

**SM:** Considering new approaches to understanding the immune response to vaccination and infection, new diseases to be controlled by vaccination, especially for emerging diseases like Ebola or Zika, therapeutic vaccines and extension to non-infectious diseases, how do you see the future of vaccinology?

**SP:** I’ve started to write and speak about this. First of all, that indeed now immunology must be the complement to vaccine development. That is to say we can no longer develop vaccines by simply counting on B cells responses and antibody responses to do the job. We are now facing diseases that are more complex where there is a risk of inducing [immune] responses that are not good and that the correlates of protection for those diseases are complex and not simply serum antibodies. I think I learned that lesson the hard way. For example, with rotavirus vaccine where the development that we went through was based really on the idea that we were developing specific antibody responses to certain viral antigens and – that is part of it – but it turns out that it is much more complicated than that. Now when we are talking about vaccines against cancer or new diseases, we are facing much more complicated situations. Maybe as a good example, there is a talk that is going to be given here on tuberculosis vaccines. It will be interesting to hear that talk because in my view, we have no idea what immune functions prevent people from getting disease in comparison to walling-off infection and remaining stable. We think that cellular immune responses are important. They may well be, but which cellular responses? It is not obvious that, let’s say, classic CD8 responses are responsible. Macrophages are key players in tuberculosis. Clearly BCG [Bacillus Calmette–Guérin] works against disseminated tuberculosis in children. But the evidence that it works to prevent adults from getting pulmonary TB [tuberculosis] is really very poor. That shows you that not only are immune functions variable, but diseases themselves may take different forms. Childhood tuberculosis is not the same as adult tuberculosis.

My point is, things are much more complicated than they used to be. Another good example is HIV because the one thing that’s worked thus far is a non-neutralising response involving natural killer cells, and nobody had predicted that. So, if we are ever going to have a vaccine against HIV, we have to think more complicated than simply developing a neutralising antibody response.

**SM:** Thank you so much for this conversation.

## Note

The *Eurosurveillance* editors acknowledge that this interview by Sanjin Musa, on the margins of a conference is not the usual form of a meeting report in *Eurosurveillance*. However, we decided to share it exceptionally as such in conjunction with the European Immunisation Week 2018.
